# Type 2 Diabetes Remission After Bariatric Surgery and Its Impact on Healthcare Costs

**DOI:** 10.1007/s11695-023-06856-0

**Published:** 2023-10-18

**Authors:** Andrew Canakis, Elizabeth Wall-Wieler, Yuki Liu, Feibi Zheng, Reem Z. Sharaiha

**Affiliations:** 1 Division of Gastroenterology and Hepatology, Department of Medicine, University of Maryland School of Medicine, 655 W. Baltimore Street, Baltimore, MD 21201 USA; 2 Global Health Economics and Outcomes Research, Intuitive Surgical, 1020 Kifer Road, Sunnyvale, CA 94086 USA; 3 DeBakey Department of Surgery, Baylor College of Medicine, One Baylor Plaza, Houston, TX 77030 USA; 4 Division of Gastroenterology and Hepatology, Department of Medicine, Weill Cornell Medicine, 1283 York Ave, 9th Floor, New York, NY 10065 USA

**Keywords:** Metabolic bariatric surgery, Obesity, Type 2 diabetes mellitus, Diabetes remission; propensity score analysis

## Abstract

**Purpose:**

Bariatric surgery is the most effective and durable treatment of obesity and can put type 2 diabetes (T2D) into remission. We aimed to examine remission rates after bariatric surgery and the impacts of post-surgical healthcare costs.

**Materials and Methods:**

Obese adults with T2D were identified in Merative™ (US employer–based retrospective claims database). Individuals who had bariatric surgery were matched 1:1 with those who did not with baseline demographic and health characteristics. Rates of remission and total healthcare costs were compared at 6–12 and 6–36 months after the index date.

**Results:**

Remission rates varied substantially by baseline T2D complexity; differences in rates at 1 year ranged from 41% for those with high-complexity T2D to 66% for those with low- to mid-complexity T2D. At 3 years, those who had bariatric surgery had 56% higher remission rates than those who did not have bariatric surgery, with differences of 73%, 59%, and 35% for those with low-, mid-, and high-complexity T2D at baseline. Healthcare costs were $3401 and $20,378 lower among those who had bariatric surgery in the 6 to 12 months and 6 to 36 months after the index date, respectively, than their matched controls. The biggest cost differences were seen among those with high-complexity T2D; those who had bariatric surgery had $26,879 lower healthcare costs in the 6 to 36 months after the index date than those who did not.

**Conclusion:**

Individuals with T2D undergoing bariatric surgery have substantially higher rates of T2D remission and lower healthcare costs.

**Graphical Abstract:**

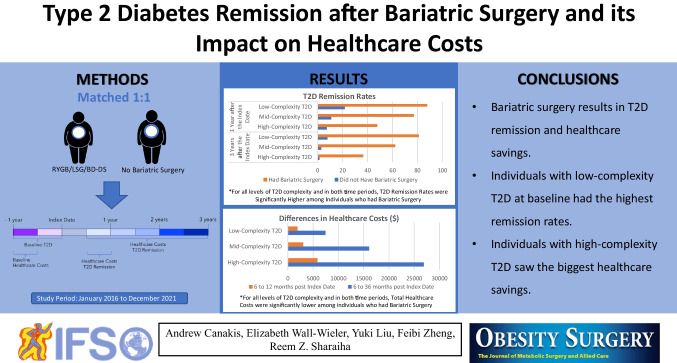

**Supplementary Information:**

The online version contains supplementary material available at 10.1007/s11695-023-06856-0.

## Introduction

Obesity is associated with a type 2 diabetes (T2D), and weight loss is associated with T2D remission among those who have T2D [1, 2]. The most effective form of weight loss is metabolic and bariatric surgery (MBS); however, rates of T2D remission vary across MBS studies [3–8]. These variations are in part due to differences in baseline obesity and comorbidity severity across studies, and variations in definitions of remission, making it difficult to compare study results. Additionally, none of these studies compares remission against individuals who did not have bariatric surgery, potentially inflating the remission rate that can be attributed to the bariatric surgery.

The remission or improvement of T2D after bariatric surgery impacts healthcare costs. Studies examining this relationship are generally conducted using economic models or healthcare claims data; those using economic models are sensitive to the inputs selected [9–12], and studies using healthcare claims data tend to compare post-bariatric surgery costs to those seen pre-bariatric surgery [13, 14]. This is a limitation as healthcare costs for those who did not have bariatric surgery would expect to increase over time, not remain at pre-bariatric surgery levels. Thus, there is still a gap in knowledge regarding the impact of MBS on healthcare costs among individuals with T2D.

As the number of MBS performed in the USA increases every year [15], the quantity of real-world data available to assess the impact of bariatric surgery on T2D increases. In this study, we examined the relationship between bariatric surgery and T2D from two perspectives: (1) does bariatric surgery result in the remission of T2D among those who have T2D at baseline; does this remission rate differ based on baseline T2D complexity? (2) Does bariatric surgery among individuals with T2D impact total healthcare costs? For each question, outcomes for individuals who had bariatric surgery were compared with matched individuals who did not have bariatric surgery.

## Methods

### Data

This study used retrospective claims data analysis from Merative™ MarketScan Research Databases (Merative™), an aggregated database that contains all paid claims and encounter data generated by more than 273 million unique patients [16]. The database includes enrollment, inpatient, outpatient, and prescription drug service use, representing the medical experience of insured employees and their dependents [16].

### Bariatric Surgery

MBS from 2016 to 2021 were examined; in this time, the most common MBS procedures were Roux-en-Y gastric bypass (RYGB), sleeve gastrectomy (SG), and biliopancreatic diversion with duodenal switch (BPD-DS) [15]. These procedures were identified in the inpatient admissions and outpatient services claims using Current Procedural Terminology (CPT)-4 and International Classification of Diseases (ICD) 10 Procedure codes (see Supplemental Table 1). For individuals who had more than one bariatric procedure identified in the study time frame, the first one was selected.

#### Baseline Type 2 Diabetes

Individuals are identified as having T2D if they have at least one diagnosis claim for T2D and have at least one diabetes-related pharmacy claim in the year before the index date. T2D diagnoses are identified in the inpatient and outpatient claims using ICD-10 diagnosis code E11 [17]. Diabetes-related pharmacy claims are identified through National Drug Codes (NDC), and were separated into three groups: metformin, antidiabetic medications (ADM), and insulin [18, 19]. ADMs include alpha-glucosidase inhibitors, amylin analogs, antidiabetic combinations, dipeptidyl peptidase-4 (DPP-4) inhibitors, glucagon-like peptide-1 (GLP-1) inhibitors, meglitinides, sodium glucose cotransporter-2 (SGLT-2) inhibitors, sulfonylureas, and thiazolidinediones.

Individuals identified as having T2D at baseline are categorized based on their disease complexity, which was defined using T2D treatment. Those who are using insulin (with or without metformin/ADM) are identified as having high-complexity T2D, those who are using at least one ADM (with or without metformin) but not using insulin are identified as having mid-complexity T2D, and those who only use metformin are identified as having low-complexity T2D.

#### Cohort

**Fig. 1:**
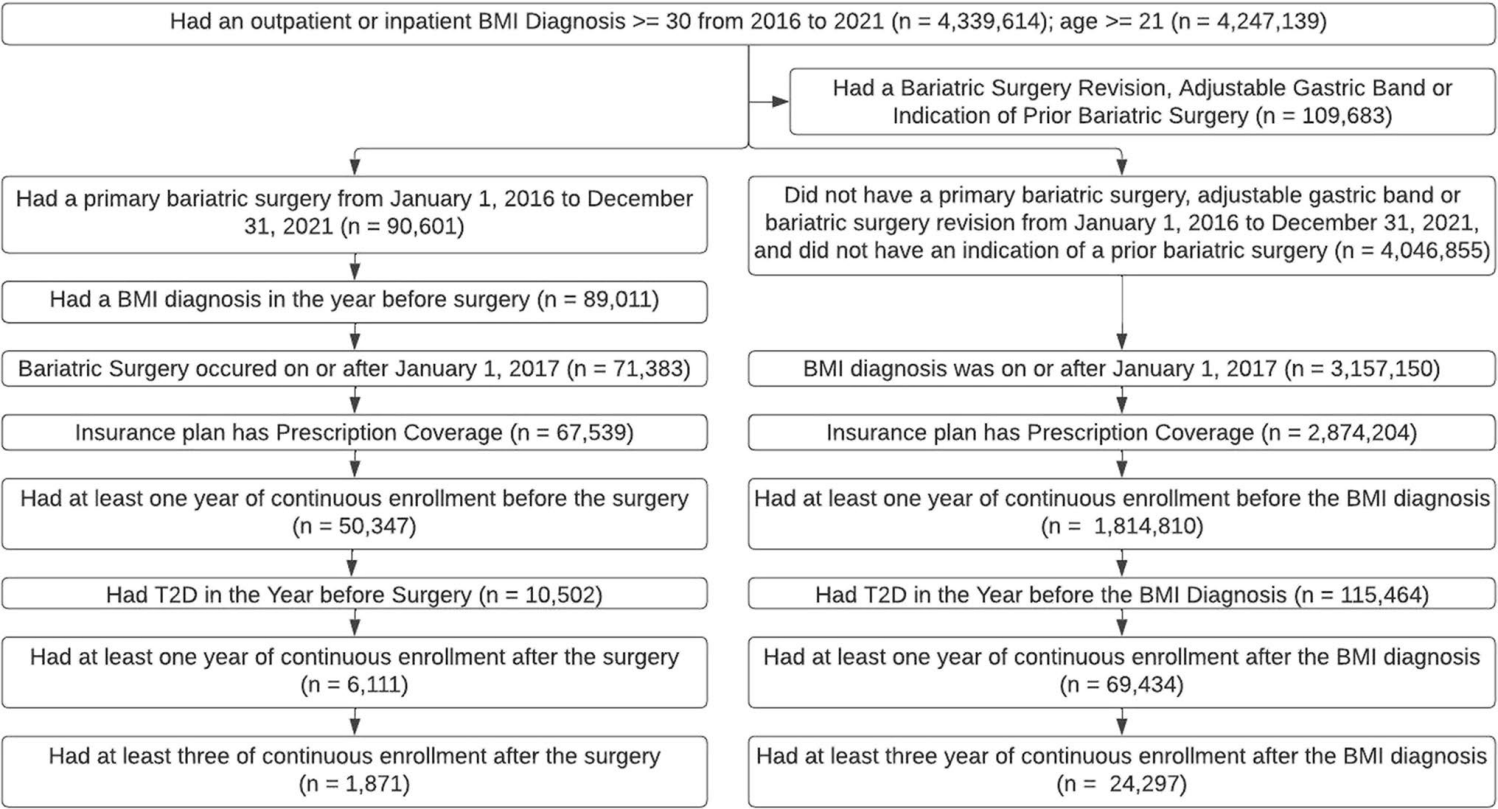
Cohort formation

This study used data from obese individuals aged 21 to 65 identified in the Merative data from January 1, 2016, to December 31, 2021. For cases, the index date is the date of bariatric surgery, and for controls, the index date is the date they had a body mass index (BMI) diagnosis; for both groups, the index date was on or after January 1, 2017 (to have at least 1 year of baseline data starting in 2016). Cases are defined as individuals who had a bariatric surgery (RYGB, LSG, BPD-DP), had a BMI diagnosis in the year before surgery, had an insurance plan with pharmaceutical coverage, were diagnosed with T2D in the year before the index date, and had at least 1 year of continuous enrollment in the year before and in the year after surgery (*n* = 6111). Controls are defined as individuals who did not have a bariatric surgery, an adjustable gastric band, a bariatric revision, or indication of a previous bariatric surgery, had an insurance plan with pharmaceutical coverage, were diagnosed with T2D in the year before the index date, and had at least 1 year of continuous enrollment in the year before and after the BMI diagnosis (*n* = 69,434). An additional analysis examined remission and new-onset T2D up to 3 years after the index data; for this analysis, there were 1871 individuals who had bariatric surgery and 24,297 individuals who did not have bariatric surgery that had at least 3 years of continuous enrollment after their index date (Fig. 1).

### T2D Remission

Among those who had T2D at baseline, we assessed T2D remission in the 6 to 12 months after the index date and in the 6–36 months after the index date. We exclude information from the first 6 months after the bariatric surgery to allow for a “wash-out” period in which prescriptions may have been used that were filled before the surgery [6]. Remission is defined as not using any diabetes-related medications in the defined time period.

### Healthcare Costs

Total healthcare costs (inpatient admissions, outpatient services, outpatient pharmaceutical claims) are examined in the 6–12 and in the 6–36 months after the index date. The first 6 months after the index date were excluded as individuals who had a bariatric surgery likely had healthcare expenses in this period related to the surgery, which do not reflect regular healthcare use. All healthcare were adjusted to 2021 constant dollars using the Medical Care component of the Bureau of Labor Statistics Consumer Price Index (http://www.bls.gov/cpi/). To remove the bias associated with extreme outliers, costs in each component (inpatient admissions, outpatient services, outpatient pharmaceutical claims) are truncated at the 1^st^ and 99^th^ percentile.

### Statistical Analysis

We first examined the characteristics of those with T2D at baseline by bariatric surgery status and then compared T2D remission at 1 and 3 years post index date for between those who did and did not have bariatric surgery.

We examined T2D remission at 1 year among those who had T2D at baseline and had at least 1 year of continuous enrollment after the index date. To compare rates of remission, those who had bariatric surgery were matched 1:1 with individuals who did not have a bariatric surgery using greedy nearest neighbor propensity scores [20]. Propensity scores were obtained using T2D severity index year, sex, age, health plan type, region, BMI, baseline healthcare costs (in the 12 to 6 months before the index date), presence of other obesity-related comorbidities (hypertension, dyslipidemia, obstructive sleep apnea, gastroesophageal reflux disease, and knee osteoarthritis—see Supplemental Table 1 for ICD-10 codes used) in the year before the index date, and whether they had an inpatient admission in the year before the index date. Balance diagnostics were examined to ensure the matched groups were balanced on all baseline characteristics; we consider a covariate to be balanced if the standardized difference is <0.25 (see Table 1; Supplemental Tables S2-S9) [21].

**Table 1:** Demographic and health characteristics among those who had T2D at baseline, by bariatric surgery status

Demographic and health characteristics	Had bariatric surgery (*N* = 6111); *n* (%)	Did not have bariatric surgery (*N* = 114,209); *n* (%)	Absolute standardized mean difference	Matched absolute standardized mean difference
Index year				
2017	2032 (33.3)	40,764 (35.7)	0.051	0.010
2018	1641 (26.9)	31,020 (27.2)	0.007	0.008
2019	1394 (22.8)	26,010 (22.8)	0.001	0.003
2020	1044 (17.1)	16,415 (14.3)	0.075	0.006
Baseline BMI; mean (SD)	42.8 (6.2)	37.1 (6.0)	0.934	0.007
Age; mean (SD)	46.5 (9.2)	52.4 (8.4)	0.667	0.040
Male sex	1940 (31.8)	57,370 (50.2)	0.383	0.013
Region				
Northeast	882 (14.4)	13,279 (11.6)	0.083	0.010
North Central	1497 (24.5)	22,890 (20.0)	0.107	0.007
South	3091 (50.6)	68,119 (59.6)	0.183	0.002
West	637 (10.4)	9817 (8.5)	0.062	0.024
Unknown	4 (0.1)	104 (0.1)	0.009	0.000
Healthcare type plan				
Comprehensive	279 (4.6)	5265 (4.6)	0.002	0.011
EPO	73 (1.2)	1125 (1.0)	0.020	0.014
HMO	671 (11.0)	14,744 (12.9)	0.060	0.008
POS	254 (4.2)	4870 (4.3)	0.005	0.002
PPO	3225 (52.8)	59,081 (51.7)	0.021	0.001
POS with capitation	65 (1.1)	1006 (0.9)	0.019	0.015
CDHP	894 (14.6)	17,876 (15.7)	0.029	0.011
HDHP	477 (7.8)	8.298 (7.3)	0.020	0.001
Obesity-related comorbidities^a^				
Hypertension	4894 (80.1)	85,351 (74.7)	0.128	0.010
Dyslipidemia	4487 (73.4)	81,926 (71.7)	0.038	0.009
OSA	3537 (57.9)	23,578 (20.6)	0.825	0.015
Knee osteoarthritis	641 (10.5)	9369 (8.2)	0.079	0.005
GERD	3433 (56.2)	16,171 (14.2)	0.980	0.063
NAFLD/NASH	1210 (19.8)	7694 (6.7)	0.392	0.050
Number of obesity-related comorbidities^a^				
1	108 (1.8)	9292 (8.1)	0.297	0.012
2	25,530 (22.4)	515 (8.4)	0.393	0.002
3+	5488 (89.8)	79,387 (69.5)	0.521	0.008
Inpatient admission^a^	492 (8.1)	10,708 (9.4)	0.047	0.010
Total healthcare costs ($)^b^; mean (sd)	10,068 (15,909)	7771 (16,462)	0.146	0.001
Baseline T2D complexity				
Low	2200 (36.0)	37,871 (33.2)	0.060	0.012
Medium	2222 (36.4)	46,975 (41.1)	0.098	0.015
High	1689 (27.5)	29,363 (25.7)	0.044	0.029

^a^In the year before the index date

^b^In the 6 to 12 months before the index date

We then compared T2D remission for those who did and did not have bariatric surgery using absolute risk differences and relative risk. Next, T2D remission at 1 year was stratified by T2D complexity, where for each complexity group, those who had bariatric surgery were matched 1:1 with those who did not have bariatric surgery based on the same characteristics as above, except for T2D severity. This analysis was replicated to examine T2D remission at 3 years among individuals who had at least 3 years of continuous enrollment after the index date.

Given the recent increased focus on bariatric surgery among Obese I and Obese Class II patients [22], we repeated each matched analysis for a sub-cohort of individuals with BMI 30–39.9.

## Results

The final cohort included 120,320 individuals who had T2D at baseline and at least 1 year of continuous enrollment after the index date, of which 6111 (5.1%) had bariatric surgery. Demographic and health characteristics varied substantially across baseline T2D and bariatric surgery status (Table 1). After propensity score matching, there were 6041 cases and 6041 controls with balanced baseline demographic and health characteristics.

### Remission of T2D

Remission of T2D at 1 year was examined among 6041 cases and 6041 controls who had at least 1 year of continuous follow-up. At 1 year, remission of T2D was 58.8 percentage points higher among those who had bariatric surgery (73.1% vs 14.2%; RR = 5.13, 95% CI 4.81, 5.47) (Table 2). When stratifying by baseline T2D complexity level and matching based on propensity scores, T2D remission rates were significantly higher among those who had bariatric surgery for each level of complexity (66.3% for low- and mid-complexity T2D, and 41% for high-complexity T2D). At 3 years post index date, 62% of individuals who had bariatric surgery were still in remission, compared to 5% of those who did not have bariatric surgery.

**Table 2:** Remission of T2D for those who did and did not have bariatric surgery

Remission time/baseline T2D	Number of matches	Remission of T2D		Risk difference (95% CI)	Relative risk (95% CI)
		Had bariatric surgery; *n* (%)	Did not have bariatric surgery; *n* (%)		
Remission, 1 year					
All	6041	4413 (73.1)	860 (14.2)	58.8 (57.4, 60.2)	5.13 (4.81, 5.47)
Low-complexity	2145	1886 (87.9)	465 (21.7)	66.3 (64.0, 68.5)	4.06 (3.74, 4.40)
Mid-complexity	2164	1666 (77.0)	235 (10.9)	66.1 (63.9, 68.3)	7.09 (6.27, 8.02)
High-complexity	1647	787 (47.8)	118 (7.2)	40.6 (37.9, 43.3)	6.67 (5.57, 7.99)
Remission, 3 years					
All	1817	1120 (61.6)	89 (4.9)	56.7 (54.3, 59.2)	12.58 (10.24, 15.46)
Low-complexity	628	509 (81.1)	49 (7.8)	73.3 (69.5, 77.0)	10.39 (7.92, 13.63)
Mid-complexity	633	394 (62.2)	18 (2.8)	59.4 (55.4, 63.4)	21.89 (13.83, 34.65)
High-complexity	508	186 (36.6)	7 (1.4)	35.2 (30.9, 39.6)	26.57 (12.62, 55.94)

### Healthcare Costs

In the 6 to 12 months and in the 6 to 36 months after the index date, those who had bariatric surgery had significantly lower healthcare costs than their matched controls who did not have bariatric surgery (Table 3). For both cases and controls, healthcare costs were lowest for those with low-complexity T2D, and highest for those with high-complexity T2D, and healthcare differences were highest among those with high-complexity T2D (were using insulin at baseline).

**Table 3:** Total healthcare costs among those who did and did not have bariatric surgery

Healthcare costs time/baseline T2D complexity	Number of matches	Total healthcare costs		Difference (*p*-value)
		Had bariatric surgery; mean (SD)	Did not have bariatric surgery; mean (SD)	
1 year				
All	6041	6771 (15,142)	10,173 (19,622)	3401 (<0.01)
Low-complexity	2145	4871 (10,774)	6768 (16,515)	1898 (<0.01)
Mid-complexity	2164	6287 (15,439)	9295 (17,584)	3007 (<0.01)
High-complexity	1647	9618 (18,534)	15,487 (21,071)	5869 (<0.01)
3 years				
All	1817	30,749 (54,516)	51,128 (64,920)	20,378 (<0.01)
Low-complexity	628	21,556 (28,199)	28,981 (33,533)	7426 (<0.01)
Mid-complexity	633	27,390 (36,086)	43,448 (43,479)	16,058 (<0.01)
High-complexity	508	45,530 (86,265)	72,408 (63,258)	26,879 (<0.01)

Finally, we looked at differences in total healthcare costs from 6 months before to 3 years after the index date. This captures all costs related to the bariatric surgery (preparation, surgery, follow-up/complications) for those who had bariatric surgery. Overall, individuals who had bariatric surgery had $3313 higher healthcare costs in this period (Table 4). Given the costs savings in the first 3 years (see Table 3), it is likely that there would be no significant difference in healthcare costs if these cohorts were followed for a few additional years. Among those who had low-complexity T2D at baseline, individuals who had bariatric surgery had substantially higher healthcare costs in this period; however, for among those who had high-complexity T2D at baseline, healthcare costs did not differ between those who did and did not have bariatric surgery.

**Table 4:** Total healthcare costs among those who did and did not have bariatric surgery, from 6 months before to 36 months after the index date

Healthcare costs time/baseline T2D complexity	Number of matches	Total healthcare costs		Difference (*p*-value)
		Had bariatric surgery; mean (SD)	Did not have bariatric surgery; mean (SD)	
3 years				
All	1817	78,384 (69.991)	75,070 (82,216)	−3313 (<0.01)
Low-complexity	628	62,734 (40,347)	44,877 (46,101)	−17,856 (<0.01)
Mid-complexity	633	74,171 (51,953)	65,920 (62,518)	−8250 (0.01)
High-complexity	508	101,424 (101,172)	105,911 (80,179)	4488 (0.44)

### Additional Analysis, BMI 30–39.9

When restricting the cohort to those with Class I and Class II obesity, there were 1596 matches with 1 year of follow-up and 473 matches with 3 years of follow-up. Remission at 1 and 3 years was very similar in the BMI 30–39 group as it was in the overall group (BMI 30+); T2D remission was 59 percentage points higher among those who had MBS (it was also 59% in the full cohort) at 1 year and 52 percentage points higher at 3 years (it was 57% in the full cohort) (Supplemental Table 2). In the BMI 30–39.9 group, the difference in healthcare costs in the 6 to 36 months after the index date was lower than in the overall cohort ($13,137 vs $20,378) (Supplemental Table 3).

## Discussion

In this large retrospective propensity score analysis of 120,320 individuals, we found that bariatric surgery is an effective and durable modality for sustaining T2D remission rates at 1 and 3 years following surgery. Regardless of T2D complexity at baseline, the surgical cohort exhibited increased remission rates with favorable cost savings as well. To our knowledge, this is the first comparative study to report these findings using a matched surgical and non-surgical cohort, and to stratify outcomes by baseline T2D complexity. Our study sheds light on the economic burden of this disease process, as T2D continues to be the leading cause of cardiovascular events and mortality worldwide [22]. These findings can help inform cost-effective medical decision, especially since the annual costs associated with a T2D individual is five times higher than those without it [23].

There is a strong relationship between obesity and T2D. MBS is the most effective and durable treatment of obesity and can put T2D into remission [24, 25]. At 1 year post-surgery, T2D remission rates range from 33 to 90% [26]. Other studies have highlighted the sustained long-term effects up to 5–10 years post-surgery [27, 28]. A recent meta-analysis of 29 studies with 4970 adolescent (with at least 5 years of follow-up) found that the long-term T2D remission rates were sustained at 90% [29]. Compared to medical therapy alone, a multitude of studies have shown that MBS is a reliable, effective, and superior option [28, 30, 31]. One randomized control trial of adults with T2D and BMI 30–45 undergoing RYGB or intensive lifestyle and medical intervention found that 1 year diabetes remission rates were 60% and 5.9%, respectively [32]. However, heterogeneity in study design and varying surgical procedures make it difficult to compare outcomes. Recognizing this knowledge gap led to the design of our study. Our data supports the effectiveness of weight loss surgery and highlights the lower healthcare costs after surgery.

Our study shows that the remission and/or improvement of T2D after MBS is associated with a reduction in healthcare costs. Studies to date have compared pre- and post-surgical outcomes. Yet this modeling is not realistic—especially since nonsurgical patients are expected to have increased costs over time. Our data highlights cost-saving measures in the surgical cohort by $3401 and $20,378 by the 6 to 12 and 6 to 36 months index dates, respectively. We also found that the higher T2D complexity equated to the largest cost difference ($26,879). Given the well-described durability of weight loss over an extended time period [28, 33], it is possible that further cost savings can be seen 6 to 10 years after surgery. A comparative retrospective study in Japan found that those undergoing bariatric surgery had significantly reduced monthly drug-related costs at 1 year, compared to those managed medically [34]. A large systemic review of 122 studies reported the cost-effectiveness of MBS versus no surgery to be $29,641/quality-adjusted life years [35]. As a clinical effective and cost effective intervention, bariatric surgery can not only reduce the complications related to T2D and obesity, but also lower medication costs for metabolic disorders and hospitalizations [36, 37]. These economic benefits superseded the costs of surgery itself [38].

The total economic burden of T2D in the USA was estimated to be $174 billion in 2007, with annual cost projected to exceed $350 billion by 2025 [23]. While bariatric surgery may impose large costs up front, its therapeutic benefit related to weight loss and other T2D complications can offset this expenditure—as seen in our study. One study found that cost savings accrued at 3 months and total laparoscopy surgery costs recovered by 26 months [38]. Yet another study found that surgery recovery costs were sustained by 5–10 years [39]. Differences in study outcomes are likely due to survey data versus simulation methods being used. In our study, we used retrospective claims data, reflecting real healthcare costs accruded by individuals over the course of their enrollment. Furthermore, monthly cost savings for diabetes medications after surgery range from 57 to 69% [40, 41].

There are a few limitations to highlight. First, there was no laboratory data available. While we were able to determine if someone filled their prescription, we were unable to verify if the individual was compliant with those medications. Inherent with any retrospective database, there also unmeasured confounders. However, the strength of this study is the robust sample size with propensity score matching and adequate long-term follow-up.

In conclusion, individuals with T2D who have bariatric surgery have substantially higher rates of T2D remission and lower healthcare costs than similar individuals who did not have bariatric surgery.

## Data Availability

The data that support the findings of this study are available from Merative(TM). Restrictions apply to the availability of these data, which were used under license for this study.

## Supplemental Materials

Table S1. Codes use to Identify Bariatric Procedures and Obesity-Related Comorbidities Supplemental Table 2: Remission of T2D for those who did and did not have Bariatric Surgery, Class I and II Obesity (BMI 30-39.9 ) Supplemental Table 3: Total Healthcare Costs among those who did and did not have Bariatric Surgery, Class I and II Obesity (BMI 30-39.9)
