# Supplementary material for: Type 2 Diabetes Remission After Bariatric Surgery and Its Impact on Healthcare Costs
Source: Obes Surg. 2023 Oct 18;33(12):3806–13. doi: 10.1007/s11695-023-06856-0 (PMC10687155; doi:10.1007/s11695-023-06856-0)
Supplement: Table S1. — Codes use to Identify Bariatric Procedures and Obesity-Related Comorbidities [file 11695_2023_6856_MOESM1_ESM.docx]

**Table S1.** Codes use to Identify Bariatric Procedures and Obesity-Related Comorbidities

| **Variable** | **Code Type** | **Codes** |
| --- | --- | --- |
| **Bariatric Surgery** |  |  |
| Roux-en-Y Gastric Bypass | CPT-4 | 43633, 43644, 43645, 43846, 43847 |
|  | ICD-10 Procedure | 0D16079, 0D1607A, 0D160J9, 0D160JA, 0D160K9, 0D160KA, 0D160Z9, 0D160ZA, 0D1607A, 0D160JA, 0D160KA, 0D160ZA, 0D1687A, 0D168JA, 0D168K9, 0D168KA, 0D168ZA, 0D168Z9, 0D16879, 0D1687A, 0D168ZA, 0D168J9, 0D16479, 0D1647A, 0D164J9, 0D164JA, 0D164K9, 0D164KA, 0D164Z9, 0D164ZA |
| Sleeve Gastrectomy | CPT-4 | 43775 |
|  | ICD-10 Procedure | 0DB64Z3, 0DQ60ZZ, 0DQ63ZZ, 0DQ67ZZ, 0DB60Z3 |
| Biliopancreatic diversion/duodenal switch | CPT-4 | 43845 |
|  | ICD-10 Procedure | 0D190Z9, 0DB60ZZ, 0DB80ZZ |
| Adjustable Gastric band | CPT-4 | 43843, 43770 |
|  | ICD-10 Procedure | 0DV64CZ |
| Bariatric Revision | CPT-4 | 43281, 43282, 43332, 43333, 43334, 43335, 43336, 43337, 43771, 43772, 43773, 43774, 43848, 43850, 43855, 43860, 43865, 43886, 43887, 43888 |
|  | ICD-10 Procedure | 0DW643Z, 0DW64CZ |
| Prior Bariatric Surgery | ICD-10 Diagnosis | Z98. 84 |
| **Obesity-Related Comorbidities** |  |  |
| Type 2 Diabetes | ICD-10 Diagnosis | E11.xx |
| Hypertension | ICD-10 Diagnosis | I10.x, I15.x |
| Dyslipidemia | ICD-10 Diagnosis | E78.x. |
| Knee Osteoarthritis | ICD-10 Diagnosis | M17.x |
| Obstructive Sleep Apnea | ICD-10 Diagnosis | G47.33 |
| Gastroesophageal Reflux Disease | ICD-10 Diagnosis | K21 x |
| NAFLD/NASH | ICD-10 Diagnosis | K75. 8, K76. 0 |
