# Supplementary material for: Type 2 Diabetes Remission After Bariatric Surgery and Its Impact on Healthcare Costs
Source: Obes Surg. 2023 Oct 18;33(12):3806–13. doi: 10.1007/s11695-023-06856-0 (PMC10687155; doi:10.1007/s11695-023-06856-0)
Supplement: Supplemental Table 2: — Remission of T2D for those who did and did not have Bariatric Surgery, Class I and II Obesity (BMI 30-39.9 ) [file 11695_2023_6856_MOESM2_ESM.docx]

**Supplemental Table 2:** Remission of T2D for those who did and did not have Bariatric Surgery, Class I and II Obesity (BMI 30-39.9 )

| **Remission time/ Baseline T2D** | **Number of Matches** | **Remission of T2D** | | **Risk Difference (95% CI)** | **Relative Risk (95% CI)** |
| --- | --- | --- | --- | --- | --- |
|  |  | **Had Bariatric Surgery; n (%)** | **Did not have Bariatric Surgery; n (%)** |  |  |
| **Remission, 1 year** | 1,596 | 1,144 (71.7) | 211 (13.2) | 58.5 (55.7, 61.2) | 5.42 (4.76, 6.17) |
| **Remission, 3 years** | 473 | 271 (57.3) | 26 (5.5) | 51.8 (46.9, 56.7) | 10.43 (7.12, 15.27) |
