# Supplementary material for: Type 2 Diabetes Remission After Bariatric Surgery and Its Impact on Healthcare Costs
Source: Obes Surg. 2023 Oct 18;33(12):3806–13. doi: 10.1007/s11695-023-06856-0 (PMC10687155; doi:10.1007/s11695-023-06856-0)
Supplement: Supplemental Table 3: — Total Healthcare Costs among those who did and did not have Bariatric Surgery, Class I and II Obesity (BMI 30-39.9) [file 11695_2023_6856_MOESM3_ESM.docx]

**Supplemental Table 3:** Total Healthcare Costs among those who did and did not have Bariatric Surgery, Class I and II Obesity (BMI 30-39.9)

| **Healthcare Costs time/ Baseline T2D Complexity** | **Number of Matches** | **Total Healthcare Costs** | | **Difference (p-value)** |
| --- | --- | --- | --- | --- |
|  |  | **Had Bariatric Surgery; mean (SD)** | **Did not have Bariatric Surgery; mean (SD)** |  |
| **1 year** | 1,596 | 7,039 (15.029) | 9,706 (18,934) | 2,667 (<0.01 |
| **3 years** | 473 | 32,553 (50,754) | 45,690 (58,921) | 13,137 (<0.01) |
